# Ethyl 4-benzamido-5-phenyl-4*H*-1,2,4-triazole-3-carboxyl­ate monohydrate

**DOI:** 10.1107/S1600536811009998

**Published:** 2011-03-23

**Authors:** Li Wang, Xue-Ying Liu, Ping-An Wang, Peng Liu, Sheng-Yong Zhang

**Affiliations:** a Department of Chemistry, School of Pharmacy, Fourth Military Medical University, Changle West Road 17, 710032 Xi-An, People’s Republic of China

## Abstract

In the title compound, C_18_H_16_N_4_O_3_·H_2_O, the dihedral angles between the triazole ring and the phenyl rings are 84.8 (4) and and 39.8 (4)°. The phenyl rings make a dihedral angle of 84.5 (9)°. In the crystal, the molecules are linked by N—H⋯O and O—H⋯N hydrogen bonds. An intra­molecular O⋯N inter­action also occurs [2.827 (3) Å]

## Related literature

For the synthesis, see: Tadha *et al.* (1973 ▶). 
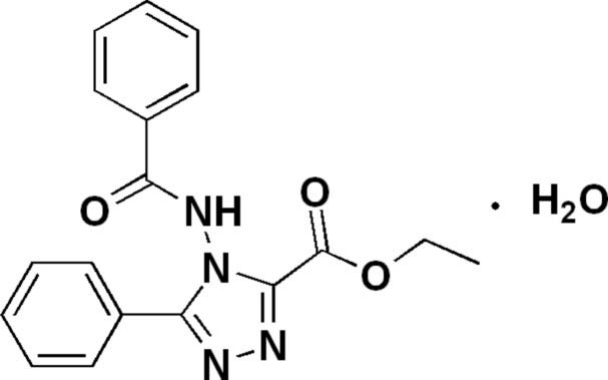


## Experimental

### 

#### Crystal data

C_18_H_16_N_4_O_3_·H_2_O
*M* *_r_* = 354.36
Triclinic, 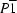

*a* = 7.932 (4) Å
*b* = 8.804 (4) Å
*c* = 13.316 (6) Å
α = 92.601 (7)°
β = 100.448 (7)°
γ = 91.378 (7)°
*V* = 913.1 (8) Å^3^
*Z* = 2
Mo *K*α radiation
μ = 0.09 mm^−1^
*T* = 296 K
0.33 × 0.28 × 0.17 mm

#### Data collection

Bruker APEXII CCD diffractometer
Absorption correction: multi-scan (*SADABS*, Bruker, 2001 ▶) *T*_min_ = 0.960, *T*_max_ = 0.979
4594 measured reflections
3197 independent reflections
2012 reflections with *I* > 2σ(*I*)
*R*_int_ = 0.022

#### Refinement

*R*[*F* ^2^ > 2σ(*F* ^2^)] = 0.062
*wR*(*F* ^2^) = 0.210
*S* = 1.02
3197 reflections
245 parameters
2 restraints
H atoms treated by a mixture of independent and constrained refinement
Δρ_max_ = 0.23 e Å^−3^
Δρ_min_ = −0.28 e Å^−3^

### 

Data collection: *APEX2* (Bruker, 2008 ▶); cell refinement: *SAINT* (Bruker, 2008 ▶); data reduction: *SAINT*; program(s) used to solve structure: *SHELXS97* (Sheldrick, 2008 ▶); program(s) used to refine structure: *SHELXL97* (Sheldrick, 2008 ▶); molecular graphics: *SHELXTL* (Sheldrick, 2008 ▶); software used to prepare material for publication: *Mercury* (Macrae *et al.*, 2006 ▶).

## Supplementary Material

Crystal structure: contains datablocks I, global. DOI: 10.1107/S1600536811009998/jh2270sup1.cif

Structure factors: contains datablocks I. DOI: 10.1107/S1600536811009998/jh2270Isup2.hkl

Additional supplementary materials:  crystallographic information; 3D view; checkCIF report

## Figures and Tables

**Table 1:** Hydrogen-bond geometry (Å, °)

*D*—H⋯*A*	*D*—H	H⋯*A*	*D*⋯*A*	*D*—H⋯*A*
N4—H4⋯O4^i^	0.86	1.99	2.776 (4)	152
O4—H4*A*⋯N1^ii^	0.83 (3)	2.17 (3)	2.990 (4)	172 (4)
O4—H4*B*⋯N2^iii^	0.82 (3)	2.09 (3)	2.893 (4)	166 (5)

Symmetry codes: (i) 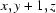; (ii) 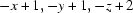; (iii) 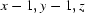.

**Fig. 1.**
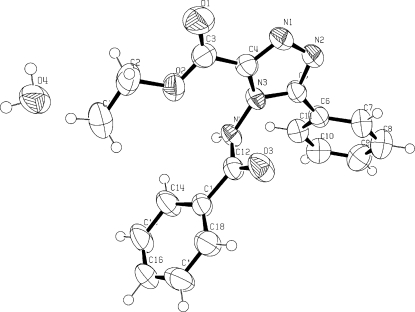
Ellipsoid plot

## supplementary crystallographic information

### Comment

l,2,4 triazole has the widespread biological activity, and it may serve as anticarcinogen, the antiviral drug, the antibacteria reagent, the fungicide,anti-inflammation agent, the analgesic and the antidepressant and so on. Some Synthetic methods had been reported about l,2,4 triazole's, but the synthetic method of the title compound C_18_H_18_N_4_O_4_, (Fig. 1) is reported for the first time. In the title compound there are the intermolecular N4—H···O2 hydrogen bonds and the intramolecular hydrogen bonds between ethyl 5-phenyl-4-[(phenylcarbonyl)amino]-4*H*-1,2,4-triazole-3-carboxylate and water. The two phenyl rings are twisted away from the plane of the triazole ring by 84.84° and 39.84° respectively. the dihedral angle between two phenyl rings is 84.59°.

### Experimental

benzohydrazide (1 equiv.)was dissolved in 100 ml toluene, then methylsulfonic acid (1 equiv.)was dropped into the solution and stirred for 20 minutes. Ethyl 2-chloro-2-oxoacetate (1 equiv.)was added subsequently and heated to reflux for 6 h until the starting material was completely consumed as monitored by TLC. The resultant residue was directly purified by flash chromatography on silica (EtOAc: Cyclohexane 1:1) gave 37% yield as a white solid. recrystallization in ethyl acetate gave fine white crystals suitable for X-ray study.

### Refinement

All H atoms were placed in idealized positions and allowed to ride on the respective parent atom with C—H distances of 0.93 (aromatic), 0.96 (CH_3_), or 0.97 (CH_2_) Å and N—H distance of 0.86 Å and with *U*_iso_(H) values of 1.2 times *U*_eq_(C)[1.5 for methyl H atoms]. 2 restraints restraints were applied.

### Crystal data

**Table:**

C_18_H_16_N_4_O_3_·H_2_O	*F*(000) = 372
*M**_r_* = 354.36	*D*_x_ = 1.289 Mg m^−^^3^
Triclinic, *P*1	Melting point: 498.450 K
*a* = 7.932 (4) Å	Mo *K*α radiation, λ = 0.71073 Å
*b* = 8.804 (4) Å	Cell parameters from 1073 reflections
*c* = 13.316 (6) Å	θ = 2.6–23.8°
α = 92.601 (7)°	µ = 0.09 mm^−^^1^
β = 100.448 (7)°	*T* = 296 K
γ = 91.378 (7)°	Block, colorless
*V* = 913.1 (8) Å^3^	0.33 × 0.28 × 0.17 mm
*Z* = 2	

### Data collection

**Table:**

Bruker APEXII CCD diffractometer	3197 independent reflections
Radiation source: fine-focus sealed tube	2012 reflections with *I* > 2σ(*I*)
graphite	*R*_int_ = 0.022
φ and ω scans	θ_max_ = 25.1°, θ_min_ = 2.3°
Absorption correction: multi-scan (*SADABS*, Bruker, 2001)	*h* = −9→9
*T*_min_ = 0.960, *T*_max_ = 0.979	*k* = −8→10
4594 measured reflections	*l* = −15→15

### Refinement

**Table:**

Refinement on *F*^2^	Primary atom site location: structure-invariant direct methods
Least-squares matrix: full	Secondary atom site location: difference Fourier map
*R*[*F*^2^ > 2σ(*F*^2^)] = 0.062	Hydrogen site location: inferred from neighbouring sites
*wR*(*F*^2^) = 0.210	H atoms treated by a mixture of independent and constrained refinement
*S* = 1.01	*w* = 1/[σ^2^(*F*_o_^2^) + (0.1284*P*)^2^] where *P* = (*F*_o_^2^ + 2*F*_c_^2^)/3
3197 reflections	(Δ/σ)_max_ < 0.001
245 parameters	Δρ_max_ = 0.23 e Å^−^^3^
2 restraints	Δρ_min_ = −0.28 e Å^−^^3^

### Special details

**Table:**

Geometry. All e.s.d.'s (except the e.s.d. in the dihedral angle between two l.s. planes) are estimated using the full covariance matrix. The cell e.s.d.'s are taken into account individually in the estimation of e.s.d.'s in distances, angles and torsion angles; correlations between e.s.d.'s in cell parameters are only used when they are defined by crystal symmetry. An approximate (isotropic) treatment of cell e.s.d.'s is used for estimating e.s.d.'s involving l.s. planes.
Refinement. Refinement of *F*^2^ against ALL reflections. The weighted *R*-factor *wR* and goodness of fit *S* are based on *F*^2^, conventional *R*-factors *R* are based on *F*, with *F* set to zero for negative *F*^2^. The threshold expression of *F*^2^ > σ(*F*^2^) is used only for calculating *R*-factors(gt) *etc*. and is not relevant to the choice of reflections for refinement. *R*-factors based on *F*^2^ are statistically about twice as large as those based on *F*, and *R*- factors based on ALL data will be even larger.

### Fractional atomic coordinates and isotropic or equivalent isotropic displacement parameters (Å^2^)

**Table:**

	*x*	*y*	*z*	*U*_iso_*/*U*_eq_	
N1	0.7002 (3)	0.9462 (3)	0.91869 (17)	0.0571 (6)	
N2	0.7312 (3)	1.0778 (3)	0.87158 (17)	0.0560 (6)	
N3	0.4588 (3)	1.0142 (2)	0.82616 (16)	0.0465 (6)	
N4	0.2941 (3)	1.0037 (2)	0.76763 (16)	0.0477 (6)	
H4	0.2113	1.0549	0.7841	0.057*	
O1	0.5395 (3)	0.7012 (3)	0.99388 (19)	0.0898 (8)	
O2	0.2954 (3)	0.7483 (2)	0.88903 (15)	0.0644 (6)	
O3	0.3957 (3)	0.8533 (2)	0.65294 (16)	0.0707 (6)	
O4	0.0919 (3)	0.1549 (3)	0.88695 (18)	0.0727 (7)	
C1	0.0303 (6)	0.6067 (6)	0.8672 (4)	0.1146 (16)	
H1A	−0.0335	0.6930	0.8831	0.172*	
H1B	−0.0248	0.5154	0.8840	0.172*	
H1C	0.0348	0.6021	0.7956	0.172*	
C2	0.2071 (5)	0.6214 (4)	0.9275 (3)	0.0797 (11)	
H2A	0.2037	0.6409	0.9993	0.096*	
H2B	0.2672	0.5281	0.9200	0.096*	
C3	0.4579 (4)	0.7737 (3)	0.9298 (2)	0.0582 (8)	
C4	0.5362 (4)	0.9084 (3)	0.88951 (19)	0.0507 (7)	
C5	0.5855 (3)	1.1164 (3)	0.81539 (19)	0.0466 (6)	
C6	0.5647 (4)	1.2538 (3)	0.75589 (19)	0.0489 (7)	
C7	0.6932 (4)	1.2967 (3)	0.7040 (2)	0.0656 (9)	
H7	0.7884	1.2369	0.7049	0.079*	
C8	0.6797 (5)	1.4283 (4)	0.6510 (3)	0.0772 (10)	
H8	0.7663	1.4574	0.6165	0.093*	
C9	0.5387 (5)	1.5165 (4)	0.6491 (3)	0.0742 (10)	
H9	0.5291	1.6046	0.6128	0.089*	
C10	0.4114 (5)	1.4739 (3)	0.7014 (2)	0.0667 (9)	
H10	0.3166	1.5343	0.7008	0.080*	
C11	0.4232 (4)	1.3429 (3)	0.7545 (2)	0.0572 (8)	
H11	0.3366	1.3144	0.7891	0.069*	
C12	0.2718 (3)	0.9074 (3)	0.6824 (2)	0.0474 (7)	
C13	0.0926 (3)	0.8745 (3)	0.6287 (2)	0.0488 (7)	
C14	−0.0503 (4)	0.9134 (4)	0.6671 (2)	0.0704 (9)	
H14	−0.0379	0.9633	0.7312	0.084*	
C15	−0.2125 (4)	0.8794 (4)	0.6118 (3)	0.0781 (10)	
H15	−0.3081	0.9072	0.6389	0.094*	
C16	−0.2340 (4)	0.8056 (4)	0.5184 (3)	0.0724 (10)	
H16	−0.3435	0.7841	0.4810	0.087*	
C17	−0.0927 (5)	0.7635 (5)	0.4803 (3)	0.0898 (12)	
H17	−0.1059	0.7109	0.4171	0.108*	
C18	0.0701 (4)	0.7985 (4)	0.5351 (2)	0.0730 (9)	
H18	0.1654	0.7700	0.5080	0.088*	
H4A	0.146 (5)	0.135 (5)	0.9435 (17)	0.107 (15)*	
H4B	−0.006 (3)	0.129 (5)	0.893 (4)	0.130 (18)*	

### Atomic displacement parameters (Å^2^)

**Table:**

	*U*^11^	*U*^22^	*U*^33^	*U*^12^	*U*^13^	*U*^23^
N1	0.0410 (14)	0.0673 (15)	0.0602 (14)	0.0028 (11)	0.0004 (10)	0.0077 (12)
N2	0.0393 (14)	0.0667 (15)	0.0608 (14)	−0.0013 (11)	0.0050 (11)	0.0079 (11)
N3	0.0313 (12)	0.0526 (12)	0.0549 (13)	0.0014 (9)	0.0061 (9)	0.0028 (10)
N4	0.0284 (12)	0.0539 (13)	0.0593 (13)	0.0032 (9)	0.0049 (9)	−0.0013 (10)
O1	0.0885 (19)	0.0843 (17)	0.0899 (17)	−0.0041 (14)	−0.0077 (14)	0.0342 (14)
O2	0.0569 (14)	0.0648 (13)	0.0733 (13)	−0.0091 (10)	0.0158 (10)	0.0125 (10)
O3	0.0417 (13)	0.0867 (15)	0.0830 (15)	0.0066 (11)	0.0149 (11)	−0.0199 (11)
O4	0.0421 (14)	0.1099 (18)	0.0658 (15)	0.0039 (13)	0.0110 (11)	−0.0049 (13)
C1	0.083 (3)	0.130 (4)	0.129 (4)	−0.045 (3)	0.016 (3)	0.027 (3)
C2	0.096 (3)	0.068 (2)	0.079 (2)	−0.0244 (19)	0.030 (2)	0.0070 (17)
C3	0.061 (2)	0.0596 (17)	0.0538 (17)	0.0004 (15)	0.0104 (14)	0.0041 (14)
C4	0.0458 (17)	0.0568 (16)	0.0495 (15)	0.0026 (13)	0.0084 (12)	0.0020 (12)
C5	0.0350 (15)	0.0524 (15)	0.0525 (15)	0.0005 (11)	0.0095 (11)	−0.0006 (12)
C6	0.0420 (16)	0.0520 (15)	0.0518 (15)	−0.0037 (12)	0.0081 (12)	−0.0020 (12)
C7	0.053 (2)	0.0646 (19)	0.084 (2)	−0.0015 (15)	0.0245 (16)	0.0067 (16)
C8	0.074 (3)	0.075 (2)	0.089 (2)	−0.0044 (19)	0.0328 (19)	0.0131 (18)
C9	0.092 (3)	0.0573 (18)	0.077 (2)	0.0009 (18)	0.0242 (19)	0.0125 (16)
C10	0.070 (2)	0.0586 (18)	0.073 (2)	0.0077 (15)	0.0145 (16)	0.0022 (15)
C11	0.0530 (19)	0.0556 (16)	0.0637 (17)	−0.0015 (14)	0.0132 (14)	0.0014 (13)
C12	0.0354 (15)	0.0519 (15)	0.0552 (16)	0.0020 (12)	0.0085 (12)	0.0046 (12)
C13	0.0373 (16)	0.0535 (15)	0.0553 (16)	−0.0016 (12)	0.0080 (12)	0.0038 (12)
C14	0.0415 (19)	0.093 (2)	0.073 (2)	−0.0067 (16)	0.0102 (15)	−0.0221 (17)
C15	0.0336 (18)	0.104 (3)	0.096 (3)	−0.0083 (16)	0.0145 (16)	−0.012 (2)
C16	0.045 (2)	0.091 (2)	0.076 (2)	−0.0186 (17)	−0.0027 (16)	0.0062 (18)
C17	0.064 (3)	0.129 (3)	0.066 (2)	−0.018 (2)	−0.0036 (18)	−0.026 (2)
C18	0.053 (2)	0.101 (2)	0.0630 (19)	−0.0017 (17)	0.0133 (15)	−0.0201 (17)

### Geometric parameters (Å, °)

**Table:**

N1—C4	1.319 (4)	C6—C7	1.385 (4)
N1—N2	1.380 (3)	C7—C8	1.380 (4)
N2—C5	1.319 (3)	C7—H7	0.9300
N3—C5	1.364 (3)	C8—C9	1.374 (5)
N3—C4	1.366 (3)	C8—H8	0.9300
N3—N4	1.393 (3)	C9—C10	1.381 (5)
N4—C12	1.368 (3)	C9—H9	0.9300
N4—H4	0.8600	C10—C11	1.376 (4)
O1—C3	1.193 (3)	C10—H10	0.9300
O2—C3	1.314 (4)	C11—H11	0.9300
O2—C2	1.464 (3)	C12—C13	1.486 (4)
O3—C12	1.222 (3)	C13—C18	1.368 (4)
O4—H4A	0.83 (3)	C13—C14	1.371 (4)
O4—H4B	0.82 (3)	C14—C15	1.380 (4)
C1—C2	1.484 (5)	C14—H14	0.9300
C1—H1A	0.9600	C15—C16	1.357 (5)
C1—H1B	0.9600	C15—H15	0.9300
C1—H1C	0.9600	C16—C17	1.364 (5)
C2—H2A	0.9700	C16—H16	0.9300
C2—H2B	0.9700	C17—C18	1.384 (5)
C3—C4	1.491 (4)	C17—H17	0.9300
C5—C6	1.472 (4)	C18—H18	0.9300
C6—C11	1.383 (4)		
			
C4—N1—N2	107.4 (2)	C8—C7—H7	120.0
C5—N2—N1	107.9 (2)	C6—C7—H7	120.0
C5—N3—C4	105.9 (2)	C9—C8—C7	120.1 (3)
C5—N3—N4	125.9 (2)	C9—C8—H8	119.9
C4—N3—N4	126.9 (2)	C7—C8—H8	119.9
C12—N4—N3	116.1 (2)	C8—C9—C10	119.7 (3)
C12—N4—H4	121.9	C8—C9—H9	120.1
N3—N4—H4	121.9	C10—C9—H9	120.1
C3—O2—C2	116.7 (2)	C11—C10—C9	120.7 (3)
H4A—O4—H4B	100 (4)	C11—C10—H10	119.7
C2—C1—H1A	109.5	C9—C10—H10	119.7
C2—C1—H1B	109.5	C10—C11—C6	119.6 (3)
H1A—C1—H1B	109.5	C10—C11—H11	120.2
C2—C1—H1C	109.5	C6—C11—H11	120.2
H1A—C1—H1C	109.5	O3—C12—N4	120.3 (2)
H1B—C1—H1C	109.5	O3—C12—C13	122.7 (3)
O2—C2—C1	107.9 (3)	N4—C12—C13	117.0 (2)
O2—C2—H2A	110.1	C18—C13—C14	118.2 (3)
C1—C2—H2A	110.1	C18—C13—C12	117.3 (3)
O2—C2—H2B	110.1	C14—C13—C12	124.5 (3)
C1—C2—H2B	110.1	C13—C14—C15	120.8 (3)
H2A—C2—H2B	108.4	C13—C14—H14	119.6
O1—C3—O2	125.7 (3)	C15—C14—H14	119.6
O1—C3—C4	121.0 (3)	C16—C15—C14	120.7 (3)
O2—C3—C4	113.3 (2)	C16—C15—H15	119.6
N1—C4—N3	109.5 (2)	C14—C15—H15	119.6
N1—C4—C3	121.3 (3)	C15—C16—C17	119.0 (3)
N3—C4—C3	129.1 (3)	C15—C16—H16	120.5
N2—C5—N3	109.2 (2)	C17—C16—H16	120.5
N2—C5—C6	124.3 (2)	C16—C17—C18	120.4 (3)
N3—C5—C6	126.4 (2)	C16—C17—H17	119.8
C11—C6—C7	119.9 (3)	C18—C17—H17	119.8
C11—C6—C5	121.1 (2)	C13—C18—C17	120.8 (3)
C7—C6—C5	118.9 (3)	C13—C18—H18	119.6
C8—C7—C6	120.0 (3)	C17—C18—H18	119.6
			
C4—N1—N2—C5	0.1 (3)	N2—C5—C6—C7	40.1 (4)
C5—N3—N4—C12	91.3 (3)	N3—C5—C6—C7	−143.7 (3)
C4—N3—N4—C12	−74.2 (3)	C11—C6—C7—C8	0.0 (4)
C3—O2—C2—C1	−176.4 (3)	C5—C6—C7—C8	−177.5 (3)
C2—O2—C3—O1	1.1 (5)	C6—C7—C8—C9	−0.3 (5)
C2—O2—C3—C4	−177.6 (2)	C7—C8—C9—C10	0.7 (5)
N2—N1—C4—N3	−1.3 (3)	C8—C9—C10—C11	−0.8 (5)
N2—N1—C4—C3	−177.7 (2)	C9—C10—C11—C6	0.4 (5)
C5—N3—C4—N1	1.9 (3)	C7—C6—C11—C10	−0.1 (4)
N4—N3—C4—N1	169.7 (2)	C5—C6—C11—C10	177.3 (2)
C5—N3—C4—C3	177.9 (3)	N3—N4—C12—O3	−10.5 (4)
N4—N3—C4—C3	−14.2 (4)	N3—N4—C12—C13	170.1 (2)
O1—C3—C4—N1	4.2 (5)	O3—C12—C13—C18	−9.5 (4)
O2—C3—C4—N1	−176.9 (3)	N4—C12—C13—C18	169.9 (2)
O1—C3—C4—N3	−171.4 (3)	O3—C12—C13—C14	169.6 (3)
O2—C3—C4—N3	7.5 (4)	N4—C12—C13—C14	−11.0 (4)
N1—N2—C5—N3	1.1 (3)	C18—C13—C14—C15	−1.3 (5)
N1—N2—C5—C6	177.9 (2)	C12—C13—C14—C15	179.6 (3)
C4—N3—C5—N2	−1.8 (3)	C13—C14—C15—C16	0.5 (6)
N4—N3—C5—N2	−169.8 (2)	C14—C15—C16—C17	0.9 (6)
C4—N3—C5—C6	−178.5 (2)	C15—C16—C17—C18	−1.5 (6)
N4—N3—C5—C6	13.5 (4)	C14—C13—C18—C17	0.7 (5)
N2—C5—C6—C11	−137.4 (3)	C12—C13—C18—C17	179.9 (3)
N3—C5—C6—C11	38.9 (4)	C16—C17—C18—C13	0.6 (6)

### Hydrogen-bond geometry (Å, °)

**Table:**

*D*—H···*A*	*D*—H	H···*A*	*D*···*A*	*D*—H···*A*
N4—H4···O4^i^	0.86	1.99	2.776 (4)	152
O4—H4A···N1^ii^	0.83 (3)	2.17 (3)	2.990 (4)	172 (4)
O4—H4B···N2^iii^	0.82 (3)	2.09 (3)	2.893 (4)	166 (5)

Symmetry codes: (i) *x*, *y*+1, *z*; (ii) −*x*+1, −*y*+1, −*z*+2; (iii) *x*−1, *y*−1, *z*.
